# Seeing the Spikes: The Future of Targetable Synthetic Voltage Sensors

**DOI:** 10.1021/acschemneuro.4c00849

**Published:** 2025-02-13

**Authors:** Tomas Fiala, David Sulzer, Dalibor Sames

**Affiliations:** † Laboratory of Organic Chemistry, ETH Zürich, D-CHAB, Vladimir-Prelog-Weg 3, 8093 Zürich, Switzerland; ‡ Department of Chemistry, Faculty of Science, Masaryk University, Kamenice 5, 625 00 Brno, Czech Republic; § Departments of Neurology, Psychiatry, and Pharmacology, Columbia University Irving Medical Center, New York, New York 10032, United States; ∥ Department of Molecular Therapeutics, New York State Psychiatric Institute, New York, New York 10032, United States; ⊥ Department of Chemistry, Columbia University, New York, New York 10027, United States; # The Zuckerman Mind Brain Behavior Institute, Columbia University, New York, New York 10027, United States

**Keywords:** voltage-sensitive dye, cell-selective targeting, membrane potential, fluorescent sensor, imaging probe

## Abstract

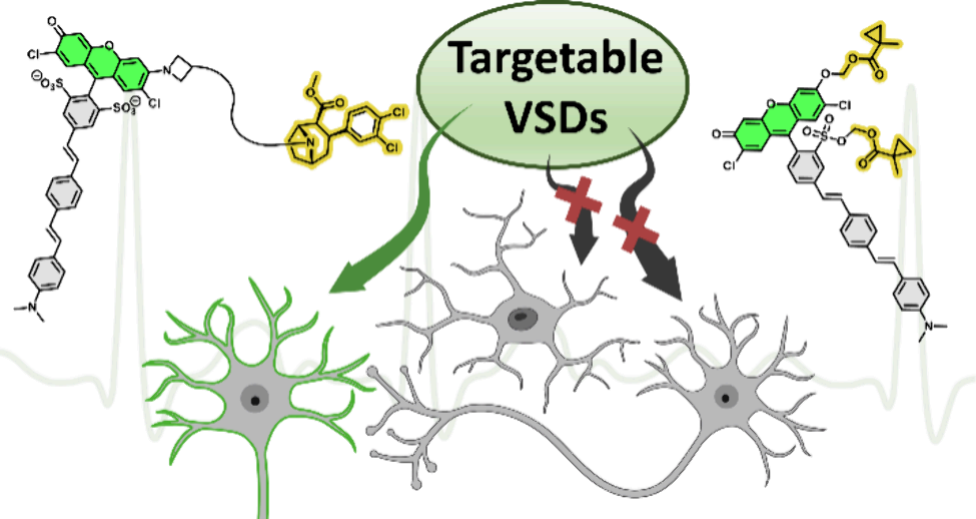

Measuring the transduction of electrical signals within neurons is a key capability in neuroscience. Fluorescent voltage sensitive dyes (VSDs) were early tools that complemented classical electrophysiology by enabling the optical recording of membrane potential changes from many cells simultaneously. Recent advances in the VSD field have led to bright and highly sensitive sensors that can be targeted to the desired cell populations in live brain tissue. Despite this progress, recently, protein-based genetically encoded voltage indicators (GEVIs) have become the go-to tools for targeted voltage imaging in complex environments. In this Perspective, we summarize progress in developing targetable VSDs, discuss areas where these synthetic sensors are or could become relevant, and outline hurdles that need to be overcome to promote the routine use of targetable VSDs in neuroscience research.

## Introduction to Synthetic Voltage Sensors

Intraneuronal signal transfer requires voltage changes at the cell membrane. Historically, most information on the membrane potential of neural cells has been gathered by electrode-based methods such as whole-cell patch-clamp electrophysiology.^1^ These techniques are low throughput, typically enabling the investigation of one, maximally a handful of cells at a time. However, elucidation of signal transfer in complex neural circuits requires a readout from hundreds of neurons at the same time. These challenges have been addressed by fluorescence-based techniques. Measuring the dynamics of intracellular calcium with optical probes has enabled the simultaneous recording of activity-dependent changes from tens to hundreds of cells.^2^ Calcium imaging, nevertheless, does not provide direct readout of membrane potential changes, lacks the temporal resolution to dissect individual depolarization events at high spiking frequencies, and cannot report on hyperpolarizing or subthreshold changes in membrane potential.^3^ These unmet needs created space for the emergence of optical electrophysiology.

**Figure 1:**
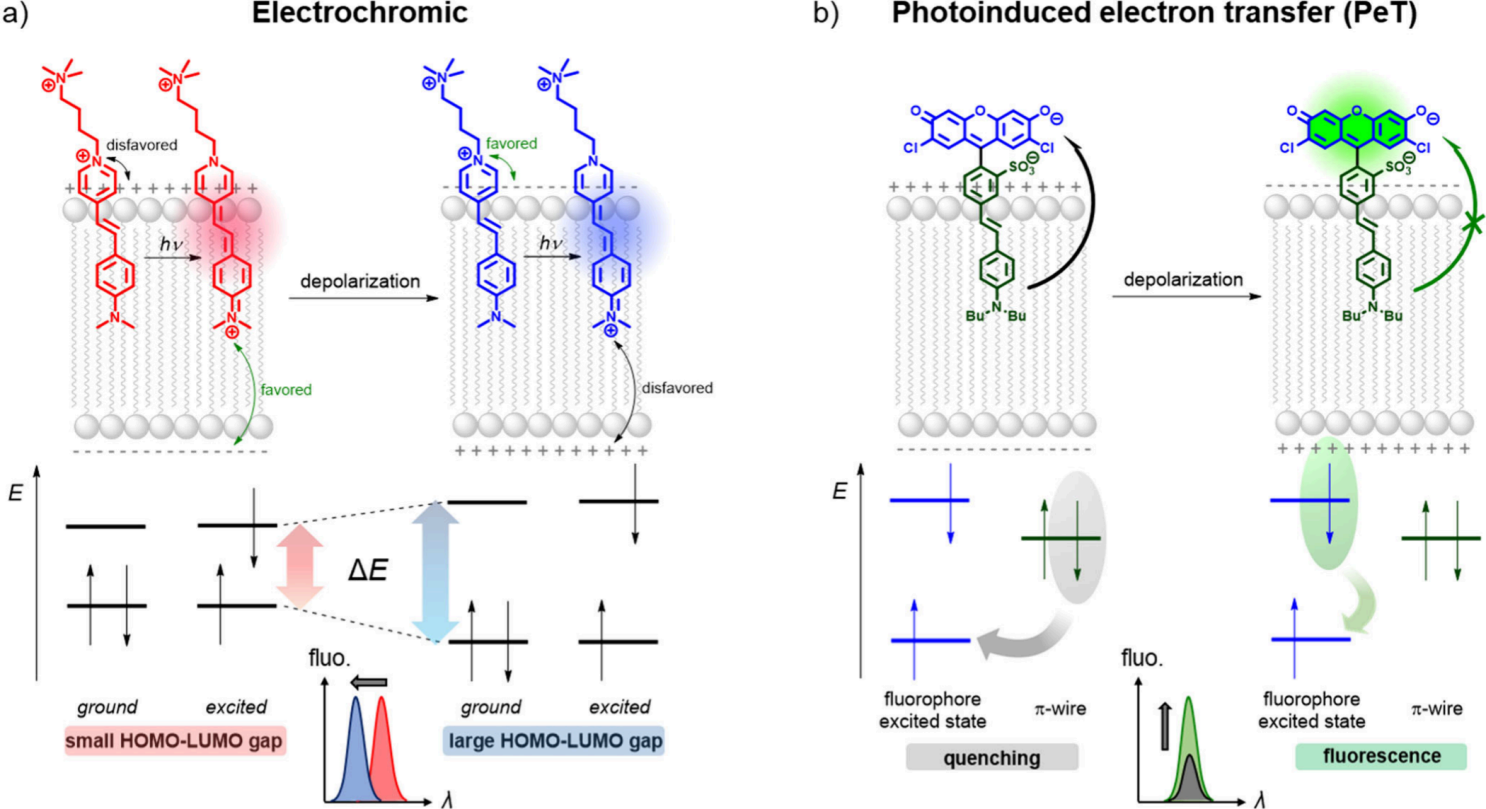
Examples of voltage sensitivity mechanisms of fluorescent dyes. (a) Electrochromic dyes: charge redistribution upon excitation of the dye causes a spectral shift upon membrane depolarization.^7^ (b) Photoinduced electron transfer (PeT)-based VSDs: membrane depolarization changes the probability of an intramolecular PeT leading to a change in fluorescence intensity.^9^

Synthetic organic fluorophores that alter their optical properties in response to changes in the surrounding electric field emerged in the 1970s.^4,5^ Over the past decades, myriad voltage-sensitive dyes (VSDs) responding to potential changes through a variety of mechanisms (reviewed in ref (6)) have been designed. The two most common classes of organic VSDs used today that show sub-millisecond responses to membrane voltage changes are electrochromic dyes^7,8^ and photoinduced electron transfer (PeT)-based dyes^9−13^ (Figure 1). These lipophilic sensors are embedded into the cell membrane, where they directly experience the uneven distribution of charges at both sides of the phospholipid bilayer. A change in membrane potential induces a change in the HOMO–LUMO gap in electrochromic dyes, thus inducing a voltage-dependent spectral shift (Figure 1a).^7^ In the case of PeT-based sensors pioneered by the Miller laboratory, the membrane potential change alters the probability of PeT from the π-wire module to the chromophore unit of the sensor, leading to a change in fluorescence quantum yield (Figure 1b).^9^

The high lipophilicity and lack of natural targeting elements of synthetic VSDs have limited the practical use of these dyes in multicellular environments. Signal originating from untargeted dyes provides only information about bulk activity.^14^ Labeling all membranes in brain tissue densely packed with neurons and non-neural cells makes the extraction of useful information challenging.^14,15^ Apart from rare cases where recording from a well-defined layer of cells is possible,^16,17^ untargeted dyes cannot provide information on cell-type-specific neuronal activity. *How can we achieve the targeting of lipophilic VSDs to tackle this challenge?*

## Targeting Voltage Sensitive Dyes

**Figure 2:**
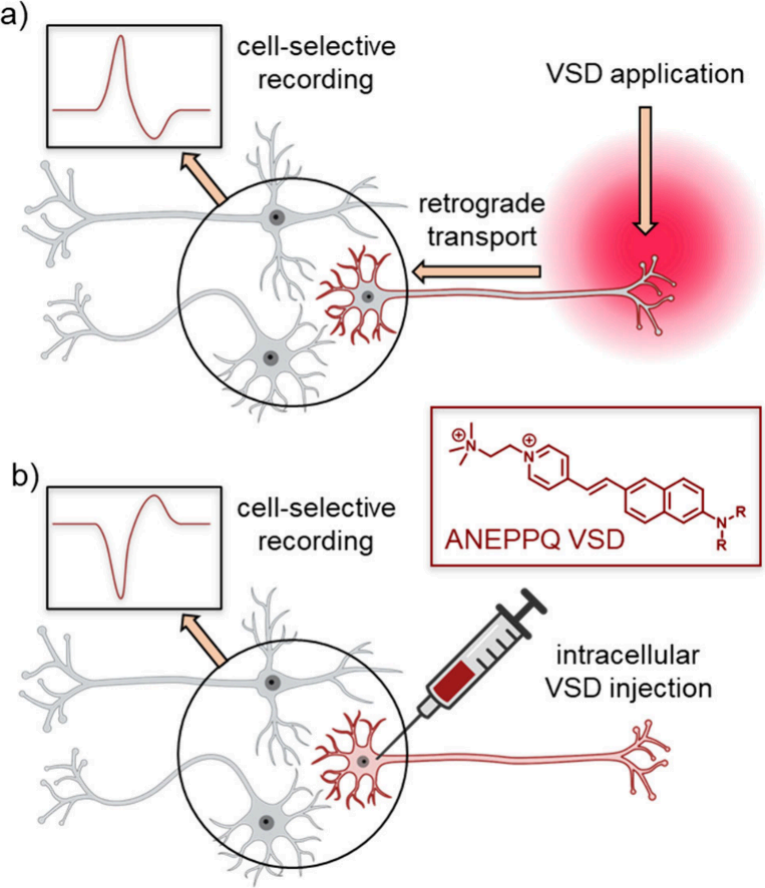
Strategies for loading unmodified VSDs into specific cell types. (a) Retrograde transport of bulk-loaded dyes along neuronal projections.^18^ (b) Direct injection into neurons.^19^

Early attempts to achieve cell-specific recordings using synthetic voltage sensors in multicellular environments took advantage of retrograde transport of bulk-loaded dyes along long neuronal projections (Figure 2a)^18^ or direct injection of VSDs into neurons (Figure 2b).^19^ However, these strategies have major limitations: the former approach cannot be used for all neuronal types, while the latter is invasive and has low throughput. The drawbacks of these cell-selective loading techniques prompted the development of VSDs carrying structural elements that enable their uncaging or immobilization at the desired target.

### Uncaging Strategies

**Figure 3:**
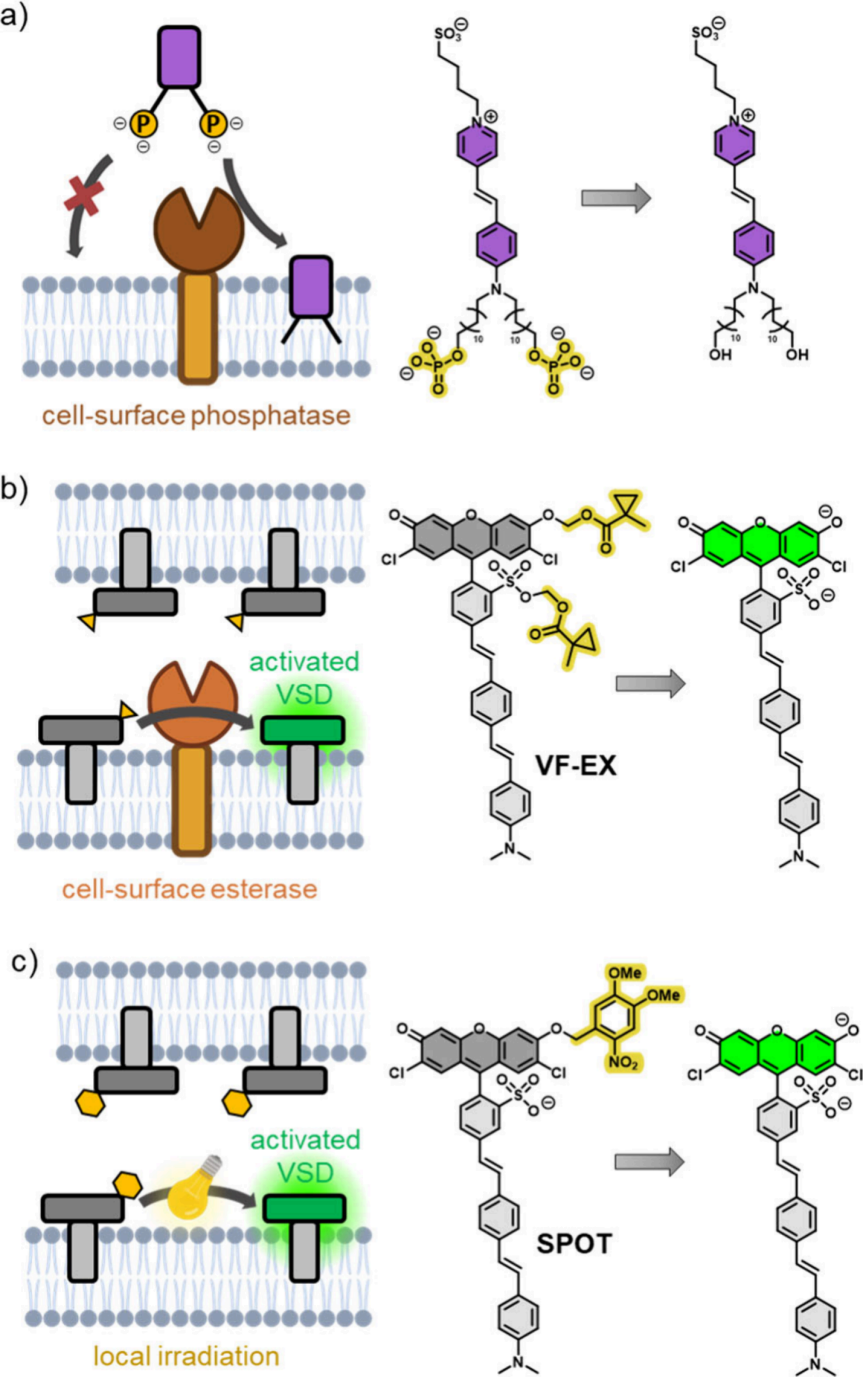
Uncaging strategies for VSD targeting. (a) Enzymatic uncaging of lipophilic anchoring groups by Hinner et al.^21^ (b) Fluorogenic enzymatic uncaging by Liu et al.^23^ (c) Fluorogenic photo-uncaging by Grenier et al.^27^

The first uncaging strategies for VSD targeting were developed by the Fromherz laboratory.^20−22^ Installation of polar phosphate ester groups into VSD headgroups^20^ or the otherwise lipophilic anchoring groups^21,22^ of an electrochromic VSD solubilized the sensor. Subsequent uncaging and selective labeling were achieved by an engineered phosphatase expressed in a cell-selective manner (Figure 3a). A similar enzymatic strategy was adopted by Liu et al.,^23^ taking advantage of fluorogenic uncaging of VoltageFluor (VF), a PeT-based VSD with a core fluorescein-type chromophore. While this “VF-EX” sensor inserts into the membranes of all cells, the action of a selectively expressed esterase transforms a dark precursor into an active VSD exclusively in target cells (Figure 3b).^23^ Ortiz et al.^24^ applied the same uncaging strategy to target improved, red-shifted voltage sensors based on the carbofluorescein chromophore. All enzymatic uncaging strategies for VSDs reported to date have used genetic engineering to express the desired enzymes in target cell types. In principle, however, the same strategy could be expanded in the future to natively expressed cell-surface or extracellular-matrix enzymes with cell-specific expression patterns.^25,26^

As an alternative to enzymatic uncaging, Grenier et al.^27^ employed light as a trigger to fluorogenically activate a PeT-based VSD (Figure 3c). In analogy to enzymatic fluorogenic uncaging, the “SPOT” voltage sensor labels all cells but becomes active in a defined location irradiated with 390 nm light.^27^ The non-selective labeling of all cells with the caged form of the VSD prior to targeted activation leads to the main drawback of these sensors, relatively low contrast between the target and non-target cells. Consequently, the use of VSD uncaging strategies has been limited to cell cultures and has yet to be demonstrated in brain tissue.

### Targeting via Self-Labeling Protein Tags

**Figure 4:**
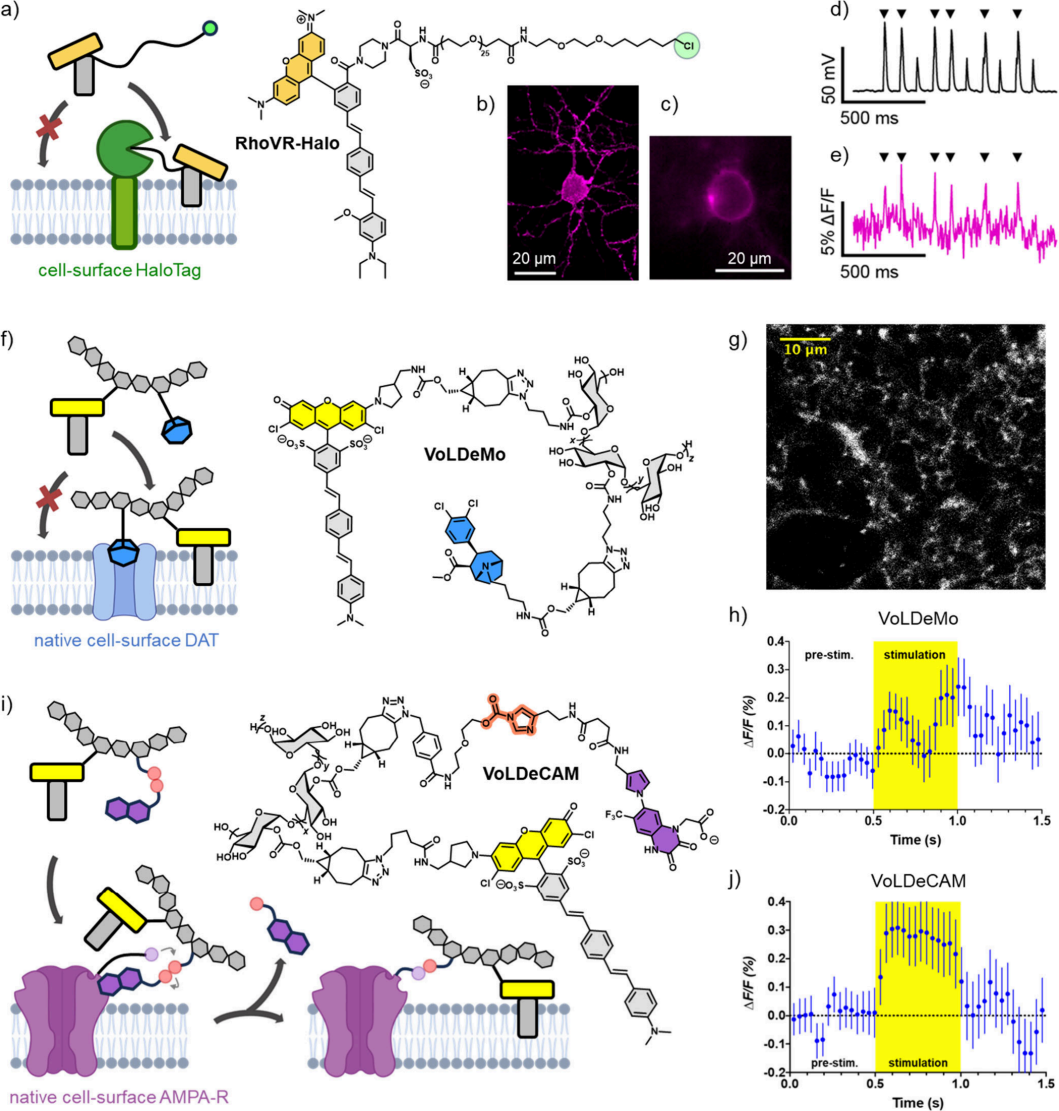
VSD targeting strategies based on binding to cell-surface proteins. (a–e) Chemogenetic targeting via a self-labeling enzyme. Example of RhoVR-Halo by Deal et al.^30^ Reproduced with permission from ref (30). (b,c) Selective labeling of HaloTag-pDisplay in mouse cortical slice by RhoVR-Halo. Two-photon (2P) fluorescence microscopy, maximum projection of 100 optical slices (b); and wide-field fluorescence microscopy (c).^30^ (d,e) Voltage imaging of action potentials evoked by current injection into the neuron in panel c. Electrophysiology trace (d), and the corresponding fluorescence trace (e). Arrows indicate evoked spikes. (f–j) Non-genetic chemical targeting strategies by Fiala et al.^33^ Reproduced with permission from ref (33). (f–h) VoLDeMo targets monoaminergic projections via a potent ligand for dopamine transporter (DAT) and norepinephrine transporter (NET). (g) Selective labeling of dopaminergic axons in mouse striatal slice by VoLDeMo. 2P fluorescence microscopy. (h) 2P voltage imaging of electrically evoked activity (25 pulses at 50 Hz) in striatal dopaminergic axons with VoLDeMo. Average of multiple trials (*N* = 100 from 10 slices). (i,j) VoLDeCAM targets neurons via a ligand for the AMPA-type glutamate receptor (AMPA-R). Ligand-directed acyl imidazole chemistry leads to covalent AMPA-R labeling and prevents long-term pharmacological perturbation. (j) 2P voltage imaging of electrically evoked activity (25 pulses at 50 Hz) in cortical neurons with VoLDeCAM. Average of multiple trials (*N* = 50 from 5 slices).

Perhaps the most successful strategy for VSD targeting so far has taken advantage of selective binding to proteins at the membrane of the target cell. Multiple examples of sensors composed of a VSD, a linker, and a reactive group that is captured by a cell-selectively expressed self-labeling enzyme have been reported (Figure 4a).^28−31^ The first example of this hybrid, chemogenetic approach came from Sundukova et al.^28^ who utilized the ACP-Tag in combination with the solvatochromic and voltage-sensitive chromophore Nile Red to measure spontaneous action potentials in cultured dorsal root ganglion sensory neurons transfected with the ACP-Tag self-labeling protein. A PeT-based VF sensor was successfully targeted by Grenier et al.^29^ via the Spy-Tag in cultured hippocampal neurons expressing the SpyCatcher protein. Utilizing HaloTag targeting, the same general strategy was employed by Deal et al.^30^ for targeting VSDs in *ex vivo* brain slices. The “RhoVR-Halo”^30^ as well as its improved, red-shifted variant “isoBeRST-Halo”^31^ allowed for the recording of single action potentials in these live brain preparations (Figure 4a-e). In addition, RhoVR-Halo was successfully employed for voltage imaging in *Drosophila* brains.^32^

### Non-Genetic Chemical Targeting

We pursued purely chemical delivery strategies for VSDs that do not rely on genetically engineered protein targets.^33,34^ Our Voltage sensor–Ligand–Dextran (VoLDe) platform combines a small dextran polysaccharide as a carrier for solubilizing the lipophilic sensor and preventing its internalization with a small molecule ligand for cell-selective targeting via binding a natively expressed protein marker of interest.^33^ With this ligand-based strategy, we achieved efficient delivery of electrochromic as well as PeT-based VSDs to dopaminergic and noradrenergic neurons in live *ex vivo* brain slices (Figure 4f,g). In addition, we successfully employed ligand-directed affinity labeling to covalently attach a VoLDe sensor to AMPA-type glutamate receptors in acute brain slices without long-term pharmacological perturbation (Figure 4i).^33^ The VoLDe platform has enabled the first cell-type-specific voltage imaging without the need for genetic manipulation (Figure 4h,j). A limitation of our approach is the relatively low signal obtained from VSDs targeted to natively expressed protein targets at their natural expression levels. Even our improved system using a brighter azetidine-functionalized dsRVF4 voltage sensor required signal averaging over multiple trials to decode electrically induced activity of the target neurons in acute mouse brain slices.^34^

### Targeting VSDs to Organelles

**Figure 5:**
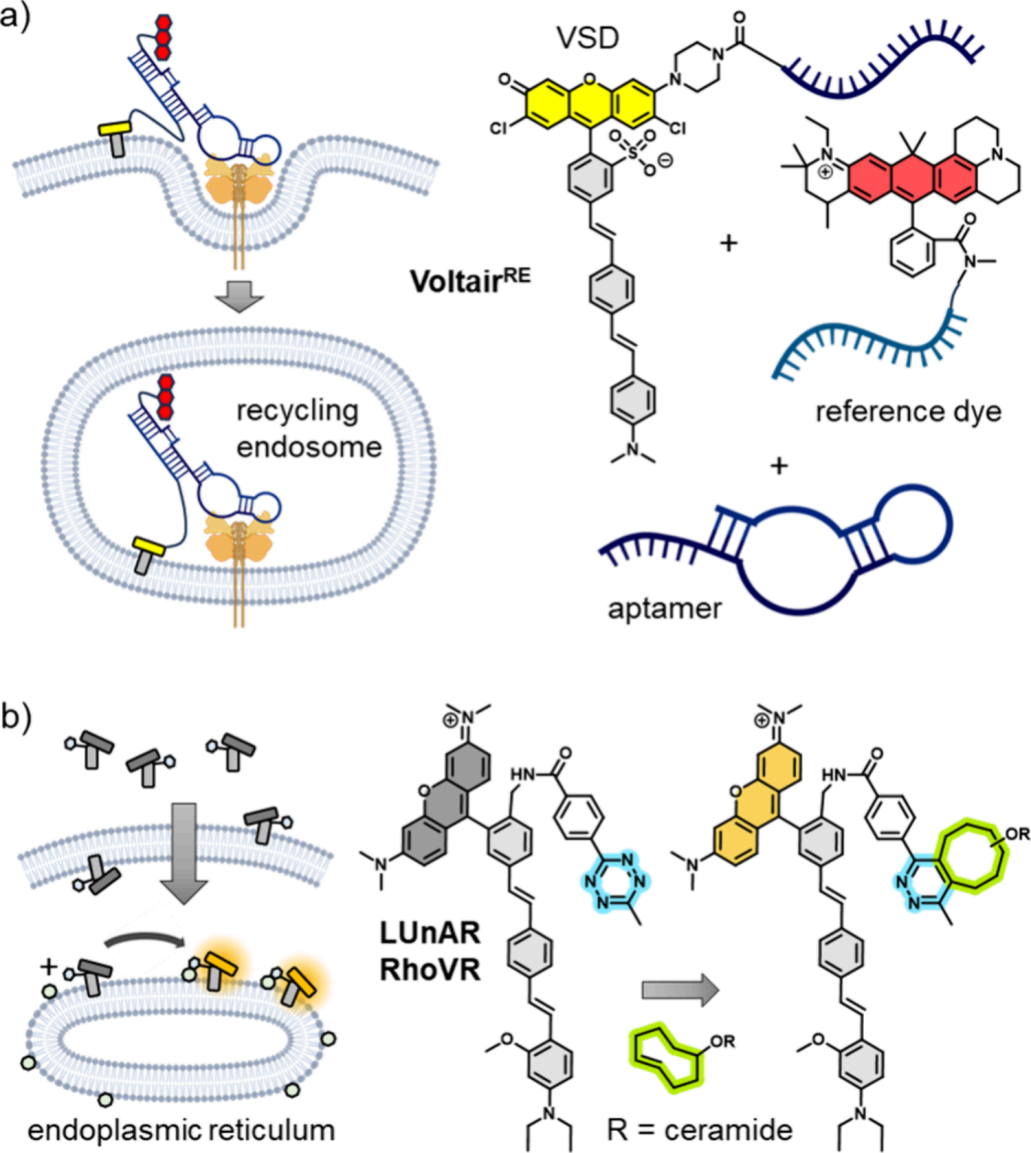
VSD targeting to organelles. (a) Voltair, a three-component DNA nanodevice by Saminathan et al.^39^ Example of Voltair^RE^ targeted to recycling endosome via an RNA aptamer for the transferrin receptor. (b) LUnAR RhoVR, a fluorogenic VSD activated by a tetrazine click reaction to *trans*-cyclooctene-functionalized ceramide in the endoplasmic reticulum by Klier et al.^40^

All of the above examples of VSD targeting focus on measuring voltage dynamics at the cell membrane of defined neuronal populations. However, an uneven charge distribution is also established at the membranes of most intracellular compartments. Historically, many cationic small-molecule dyes were identified that accumulate in the mitochondrial matrix in a voltage-dependent manner and thus report on changes in mitochondrial membrane potential.^35^ Improved methods for mitochondrial voltage imaging that use modern dyes^36,37^ and targeting, e.g., via esterase-mediated unmasking^37,38^ have been recently developed. There has been growing interest lately in developing strategies to target VSDs to defined organelles beyond mitochondria, implementing state-of-the-art targeting strategies. Saminathan et al.^39^ have developed “Voltair”, a nucleic acid-based nanodevice for measuring the membrane potential of endosomes, lysosomes, and the trans-Golgi network in cultured HEK 293T cells with a PeT-based VSD (Figure 5a). Adopting a fluorogenic strategy, Klier et al.^40^ developed “LUnAR RhoVR”, a tetrazine-substituted PeT-based sensor that is activated by undergoing a click reaction with *trans*-cyclooctene-functionalized ceramide in the endoplasmic reticulum (ER) (Figure 5b). This sensor provided the first evidence for the functional coupling between the membrane potentials at the plasma membrane and the ER in cultured HeLa cells.^40^

### VSD Targeting: What Now?

Despite the many innovations in targeted voltage imaging with synthetic VSDs, state-of-the-art sensors have yet to be successfully applied in truly complex *in vivo* environments. Current methodologies have fallen short of demonstrating utility beyond proof-of-concept recordings in *ex vivo* rodent brain slices.^30,31,33,34^ What factors have so far prevented the successful use of synthetic VSDs for targeted voltage imaging *in vivo*? *Is there even a place for synthetic sensors in a field dominated by protein-based reporters?*

## Synthetic Voltage Sensors in the Era of Genetically Encoded Indicators

In contrast to synthetic VSDs, genetically encoded voltage indicators (GEVIs) are now routinely used for targeted voltage imaging *in vivo* (reviewed in refs (3 and 41−43)). GEVIs are proteins derived from a voltage-sensitive protein domain that are engineered to transform the voltage response to a fluorescent signal, via either intrinsic fluorescence or coupling to a fluorescent protein or a small-molecule fluorophore.^43−45^ Early GEVI designs suffered from major drawbacks, such as imperfect sensor trafficking to the cell membrane, creating additional membrane capacitance, or passing photocurrents, all of which perturb the system under study. In addition, some modern opsin-based GEVIs (such as paQuasAr3) require photoactivation with blue light to achieve good signal-to-noise ratio (SNR).^46^ Despite these shortcomings which persist to date, dramatic improvements in design now enable state-of-the-art GEVIs to address key neuroscience questions at various anatomic levels *in vivo*, including large brain regions,^47^ neural circuits,^46^ populations of individual cells,^48^ or even subcellular regions.^46^ Such insights are inaccessible to calcium imaging or electrophysiology. Given the success of GEVIs for *in vivo* voltage imaging, we need to ask what motivations remain to further develop synthetic VSDs for targeted imaging in live brain tissue.

### Do We Still Need Targetable Synthetic VSDs?

We see several arguments that could support further efforts to develop synthetic sensors for targeted voltage imaging.

**Figure 6:**
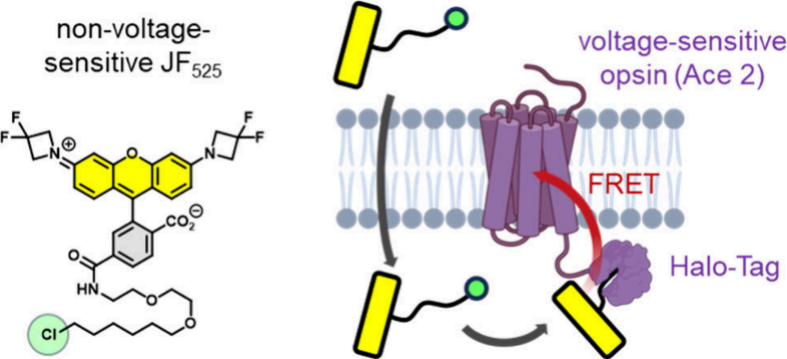
Voltron, a hybrid chemogenetic GEVI composed of a voltage-sensitive opsin fused to a Halo-Tag self-labeling enzyme that captures a non-voltage-sensitive fluorescent dye. Voltage-dependent FRET between the dye and the opsin enables optical readout of membrane potential changes.^44^

1. *Superiority of synthetic dyes.* A long-standing argument for the development of VSDs has been the superiority of synthetic dyes to fluorescent proteins. Organic chemistry provides a virtually unlimited structural space to fine-tune the photophysical and chemical properties of dyes, including absorption and emission wavelengths, Stokes shift, photostability, brightness, two-photon cross section, voltage sensitivity, polarity, and responsiveness to other environmental factors (such as pH). In contrast, mutating the sensor domains of GEVIs (opsins or fluorescent proteins) does not provide such flexibility. Nevertheless, this argument has been weakened in recent years. Optimization of fluorescent proteins yielded bright and photostable reporters with red to near-infrared emission that have been successfully incorporated into GEVIs.^49^ In addition, recently developed hybrid chemogenetic GEVIs that link small-molecule non-voltage-sensitive fluorophores to voltage-sensitive opsins combine the advantages of both synthetic dyes and protein-based voltage sensors.^44,45,50^ For instance, “Voltron” sensors^44,45^ use a HaloTag approach to immobilize a Janelia Fluor (JF) dye in the vicinity of the opsin and respond to membrane potential via a Förster resonance energy transfer (FRET) mechanism (Figure 6). Since simple HaloTag fluorophores can be optimized for high blood–brain barrier (BBB) and cell penetration, this approach has been highly successful even for *in vivo* voltage imaging in mice, zebrafish, and fruit flies and represents the current state-of-the-art.^44,45^ In contrast, BBB penetration of targetable VSDs that contain lipophilic dyes fused to long solubilizing linkers is yet to be demonstrated and might represent a challenge for their systemic administration.
2. *Ready-to-go tools.* GEVIs require genetic manipulation of the system under study. Especially for *ex vivo* or *in vivo* applications, genetic manipulation can represent a laborious and time-consuming task. For instance, good expression of the protein of interest in the mouse brain after viral transfection with common viral vectors is typically achieved after at least 3–4 weeks.^51^ For studies in models of brain disorders which are already based on multiple genetic modifications, further genetic manipulation is impractical. Probes that utilize non-genetic targeting strategies are particularly appealing alternatives to genetic methods that can be readily employed without long preparation of the model system. We previously introduced fluorescent false neurotransmitters (FFNs) as small-molecule non-genetic tracers of catecholamines in the brain.^52,53^ FFNs have become widely used in a broad range of experimental settings, including *in vivo*, and complement genetically encoded neurotransmitter sensors.^54^ In analogy, future development of non-genetically targeted VSDs^33,34^ could yield ready-to-use sensors complementary to state-of-the-art GEVIs.
3. *Voltage imaging in systems with unexplored genetics.* Methods for the genetic manipulation of standard model organisms such as rodents, zebrafish, or fruit flies are well established. However, beyond these common systems, obtaining transgenic species is highly challenging. Well below 1% of all eukaryotes have been sequenced so far.^55^ This means that genetically encoded sensors cannot be straightforwardly used in over 99% of species. In contrast, purely chemical probes that recognize protein targets known to be highly conserved can in principle be readily employed in a broad range of organisms. In case of a need to record membrane potential changes in an organism outside of the research mainstream, synthetic VSDs are the only immediate option available.
4. *Molecule-specific targeting for localized voltage imaging.* As early as the 1980s, Loew and co-workers showed using synthetic VSDs that external stimuli such as an electric field may trigger a nonuniform membrane potential of cells.^56^ Localized changes of membrane potential in specialized protrusions of the cell membrane, including primary cilia^57^ or dendritic spines,^58,59^ are now believed to play key biological roles in cell division and synaptic function, respectively. Compared to microdomains of intracellular calcium,^60^ however, electrical compartmentalization at cellular membranes remains underexplored. Targetable synthetic VSDs are well-suited for voltage sensing at defined locations of the cell membrane. In analogy to targetable calcium-sensitive dyes,^61^ VSDs could be delivered to the vicinity of selected ion channels via small chemogenetic tags^61^ or traceless affinity labeling^33^ to record local membrane potential changes. Small synthetic probes are less likely to perturb the native function of these channels compared to fusion with large protein-based GEVIs.

In addition to the reasons mentioned above that warrant continued development of targetable VSDs, it is generally wise for the scientific community to explore diverse approaches to a given problem. We therefore ask: *what are the main issues that need to be overcome to bring targetable synthetic voltage sensors to the mainstream?*

## Issues to Overcome

A great obstacle that hinders the widespread use of targetable VSDs is their low sensitivity in complex experimental systems. In *ex vivo* rodent brain slices, the state-of-the-art chemogenetically targeted RhoVR-Halo and isoBeRST-Halo provided single action potential recordings but with a suboptimal SNR (Figure 4e).^30,31^ In a similar system, our VoLDe probes chemically targeted to natively expressed protein targets provided useful readout only after signal averaging from multiple trials (Figure 4h,j).^33,34^ As a consequence, targeted VSDs have not been employed in live mammalian brain tissue beyond these proof-of-concept studies.

**Figure 7:**
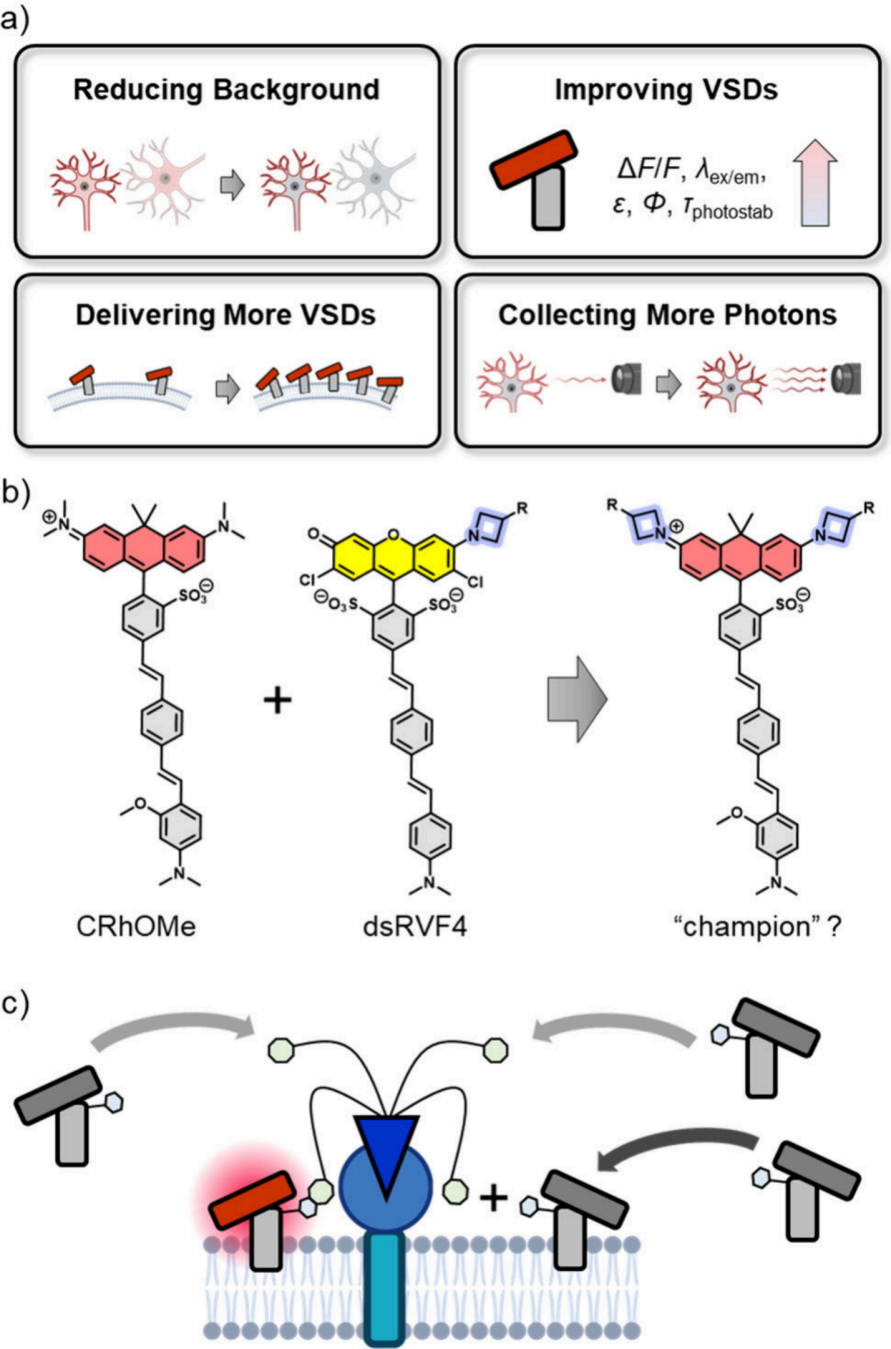
Outlook for targetable synthetic VSDs. (a) Four strategies for improving voltage imaging in tissue. (b) Combining structural features that improve VSD performance: fusing the red-shifted CRhOMe sensor^63^ with the bright, azetidine-functionalized dsRVF4 (ref (34)) could provide an improved VSD suitable for targeted voltage imaging. R = H, F, linker. (c) Potential strategy to deliver more VSDs to target cells: a polyfunctional, dendrimeric delivery vehicle that captures multiple VSDs per single target.

The SNR in optical electrophysiology depends primarily on two parameters. It scales linearly with the voltage sensitivity of the sensor (fluorescence change over basal fluorescence, Δ*F*/*F*) and with the square root of the number of collected photons.^62^ Superior voltage imaging should thus be achievable with (1) lower background fluorescence; (2) brighter or more sensitive VSDs; (3) a higher number of delivered VSDs; and (4) improved photon collection (Figure 7a).

### Can We Reduce Background Signal?

In complex environments of live tissue, the measured Δ*F*/*F* response of any sensor is almost always lower than that *in vitro*. This is in part due to a higher background in tissue from autofluorescence or misdelivered sensors. The former can be mitigated by design of sensors operating at longer wavelengths where the intrinsic fluorescence of biomolecules is minimal. For the latter, although great progress has been made in VSD targeting, the high lipophilicity of synthetic voltage sensors resulting in significant background labeling remains a challenge. New delivery vehicle designs are thus needed to provide more selective neuronal labeling with VSDs.

### Can We Design Better VSDs?

In recent years, multiple groups have contributed important insights into the design of VSDs as well as brighter and more photostable fluorophores, in general. In one promising advance, Gest et al.^63^ have introduced “CRhOMe”, the most advanced PeT-based voltage sensor based on carborhodamine. We have previously reported that azetidine substitution provides brighter and more sensitive PeT-based VSDs.^34^ While the field has not yet produced a VSD “champion”, combining these designs could lead to an improved sensor suitable for targeted voltage sensing in challenging environments (Figure 7b).

Apart from PeT-based VSDs, the photophysical properties (e.g., absorption and emission wavelength, brightness) of the highly sensitive ANNINE-type electrochromic sensors promise room for optimization, although these sensors are yet to be transformed into targetable probes.^64^ In addition, red-shifted cyanine-based dyes have received growing attention in the chemical biology field^65^ but remain underexplored as voltage sensors.^66^ We thus see ample potential for further development of brighter and more sensitive VSDs in the near future.

### Can We Deliver More VSDs?

Direct comparison of the performance of chemogenetically targeted VSDs to protein-based GEVIs in cultured neurons favors some of the synthetic sensors.^23,29,32^ However, this advantage has not been successfully translated into complex *in vivo* systems. Is the number of delivered sensors to blame? The quantity of delivered VSDs is tied to the expression levels of their molecular targets, which is typically enhanced by strong promoters.^30,31^ In the future, even stronger promoters could further promote VSD delivery for improved voltage imaging in brain tissue. However, a fundamental question arises: what are the expression levels of these “overexpressed” targets? Good expression is often assessed only qualitatively^30^ and the number of delivered sensors per cell or unit membrane area is almost never determined. Therefore, even when the performance of synthetic VSDs and GEVIs is experimentally compared, one needs to be careful in interpreting the results if the numbers of delivered sensors are not comparable or are unknown.^23^

Manipulating protein expression levels is not possible for VSDs that target native protein targets.^33,34^ For these systems, future research could focus on catalytic sensor delivery. Established approaches, such as dye uncaging with native extracellular enzymes^26^ (*vide supra*, Figure 3) or hijacking native transporters^67^ could be adopted for VSD targeting. In addition, polyfunctional vehicles such as dendrimers^68^ could allow for the delivery of multiple VSDs per target (Figure 7c).

### Can We Collect More Photons?

To resolve individual action potentials, we need to image the relatively small, quasi-two-dimensional cell membrane rather than the full cell volume with millisecond temporal resolution. These requirements greatly restrict the number of collected photons, leading to poor SNR. Recent efforts have brought significant improvements in kilohertz two-photon imaging techniques that combine the high spatial resolution from two-photon excitation and rapid imaging rates,^69,70^ as well as VSDs with red or near-infrared absorption/emission that penetrates efficiently through tissue.^31,63^ Further development of such techniques and probes plus lasers, optics, and detectors optimized for long wavelengths will be crucial for efficient photon collection and overall high-fidelity voltage imaging with targetable VSDs.

## Summary and Outlook

Here, we summarize strategies for targeted voltage imaging with synthetic VSDs, the challenges the field faces, and possible future developments to improve these sensors. While impressive advances in VSD design and delivery to specific neuronal populations in brain tissue have been achieved, synthetic sensors are currently being overshadowed by genetically encoded sensors. We outline areas, such as the study of organisms with unexplored genetics or molecule-specific targeting for localized voltage imaging, where we believe that targetable synthetic VSDs could find applications. Finally, we discuss advances, including the design of better delivery vehicles and VSDs, the delivery of more VSDs, and more efficient photon collection, that are necessary prerequisites to bring these sensors to the neuroscience mainstream. A concerted effort at multiple fronts and democratizing access to state-of-the-art probes as well as instruments, e.g., via large government initiatives such as the National Institutes of Health BRAIN initiative or the proposed National Brain Observatory, will be key for the advancement of targeted voltage imaging and neuroscience at large.^62^

## Dedication

Dedicated to Prof. Vladimír Šindelář on the occasion of his 50th birthday.
